# Risk Factors for Maternal Mortality in Rural Tigray, Northern Ethiopia: A Case-Control Study

**DOI:** 10.1371/journal.pone.0144975

**Published:** 2015-12-17

**Authors:** Hagos Godefay, Peter Byass, Wendy J. Graham, John Kinsman, Afework Mulugeta

**Affiliations:** 1 Tigray Regional Health Bureau, Mekelle, Ethiopia; 2 Umeå Centre for Global Health Research, Department of Public Health and Clinical Medicine, Umeå University 90187 Umeå, Sweden; 3 Institute of Applied Health Sciences, School of Medicine and Dentistry, University of Aberdeen, Aberdeen, United Kingdom; 4 MRC/Wits Rural Public Health and Health Transitions Research Unit, School of Public Health, Faculty of Health Sciences, University of the Witwatersrand, Johannesburg, South Africa; 5 College of Health Sciences, Mekelle University, Mekelle, Ethiopia; Harvard Medical School, UNITED STATES

## Abstract

**Background:**

Maternal mortality continues to have devastating impacts in many societies, where it constitutes a leading cause of death, and thus remains a core issue in international development. Nevertheless, individual determinants of maternal mortality are often unclear and subject to local variation. This study aims to characterise individual risk factors for maternal mortality in Tigray, Ethiopia.

**Methods:**

A community-based case-control study was conducted, with 62 cases and 248 controls from six randomly-selected rural districts. All maternal deaths between May 2012 and September 2013 were recruited as cases and a random sample of mothers who delivered in the same communities within the same time period were taken as controls. Multiple logistic regression was used to identify independent determinants of maternal mortality.

**Results:**

Four independent individual risk factors, significantly associated with maternal death, emerged. Women who were not members of the voluntary Women’s Development Army were more likely to experience maternal death (OR 2.07, 95% CI 1.04–4.11), as were women whose husbands or partners had below-median scores for involvement during pregnancy (OR 2.19, 95% CI 1.14–4.18). Women with a pre-existing history of other illness were also at increased risk (OR 5.58, 95% CI 2.17–14.30), as were those who had never used contraceptives (OR 2.58, 95% CI 1.37–4.85). Previous pregnancy complications, a below-median number of antenatal care visits and a woman’s lack of involvement in health care decision making were significant bivariable risks that were not significant in the multivariable model.

**Conclusions:**

The findings suggest that interventions aimed at reducing maternal mortality need to focus on encouraging membership of the Women’s Development Army, enhancing husbands’ involvement in maternal health services, improving linkages between maternity care and other disease-specific programmes and ensuring that women with previous illnesses or non-users of contraceptive services are identified and followed-up as being at increased risk during pregnancy and childbirth.

## Introduction

Maternal mortality—which persists at unacceptably high levels in all of sub-Saharan Africa [1]–is determined by a wide range of factors including, among others, individual women’s circumstances and characteristics, logistic support in the event of emergencies, and health service availability and quality. Many of these factors tend to be inter-correlated, and careful investigation is needed to tease out which factors are major, and potentially modifiable, determinants.

Globally, maternal mortality ratio fell by 45% between 1990 and 2013, with a slightly faster fall of 49% in sub-Saharan Africa [2]. This means that the overall aim of Millennium Development Goal 5 (MDG5), a 75% reduction in mortality, is very unlikely to be achieved on a wide scale by the end of 2015. High levels of mortality continue, particularly in low-resource regions such as sub-Saharan Africa, despite widespread recognition of women’s rights to the highest standards of maternity care [3]. There have been substantial maternal mortality reductions in many regions of the world outside sub-Saharan Africa, but areas with persistently high mortality have been described as “one of the shameful failures of development” [4]. In Ethiopia, despite marked improvements in the utilisation of maternal health services, and an estimated fall of 69% in the WHO estimates of maternal mortality from 1990 to 2013, rates of maternal mortality remain high [5].

The lack of sound and comprehensive evidence on maternal mortality often hampers the implementation of appropriate health policies and interventions to counter women’s deaths. In order to develop, implement and evaluate policies for reducing maternal mortality, it is essential to understand the associated risk factors. National sample surveys such as the Demographic and Health Survey (DHS) [6] provide some information on maternal risks in many low- and middle-income countries, but the retrospective nature of the birth history approach and relatively small sample size limits its scope. For Ethiopia, there have been specific efforts to analyse the maternal findings of successive DHS rounds [7]. In the industrialised world, although better quality data may be available, there are very small absolute numbers of maternal deaths to analyse epidemiologically. Maternal death has been linked to inadequate uptake of antenatal care, previous medical conditions and previous pregnancy complications in the UK. [8] In Brazil, maternal death was associated with lower maternal education and lack of antenatal care as well as previous Caesarean sections. [9]

There have been specific studies of risk factors associated with maternal death in Ethiopia [10–12], mostly facility-based and conducted in urban settings. In Ethiopia, as the majority of deliveries and maternal deaths occur at home (estimated at around 90% in the 2011 DHS survey [6]), results from facility-based studies might not accurately reflect risk factors for maternal death as a whole or for those that occur outside health facilities. Our previous work has shown massive variations in community-based maternal mortality in Tigray Region across relatively small geographic distances, which could not reasonably be accounted for by risk factors pertaining to individual women [5]. The aim of this study is to characterise individual determinants of maternal mortality in the particular setting of Tigray Region, Ethiopia, irrespective of geographical variations.

## Methods

### Study setting

**Fig 1:**
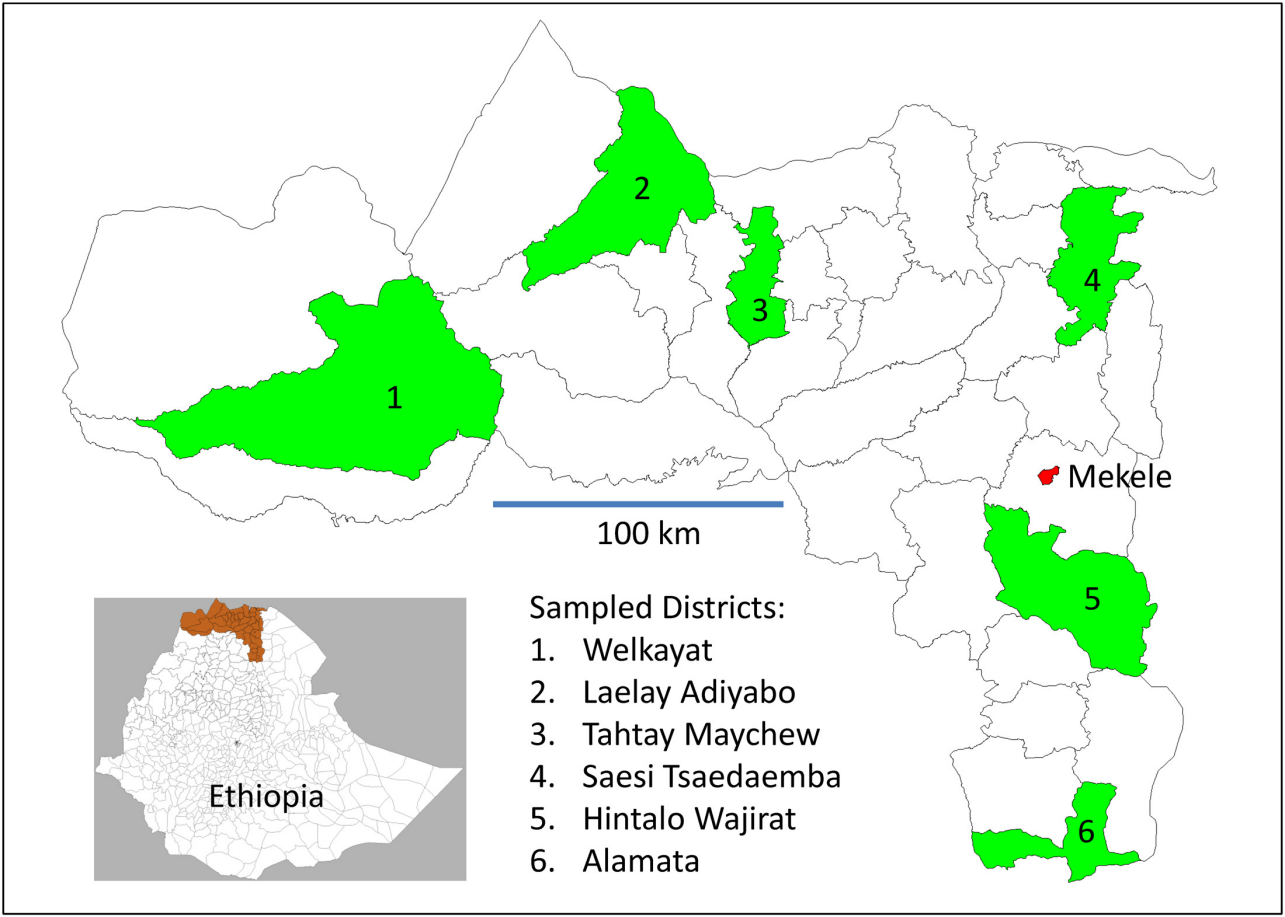
Location of study districts in Tigray Regional state, Northern Ethiopia (from Godefay et al. Global Health Action 2014; 7:25264 [13]).

Tigray Region is located in northern Ethiopia, with a total population of more than 5.1 million. Details of the geography and population in the Region are described in more detail in a previous paper [5]. Most of the population live in scattered rural villages, some of which are quite remote in terms of access and facilities. As previously described [13], a study of maternal mortality was conducted in six rural districts of Tigray Region (Welkayat, Laelay Adiyabo, Tahtay Maychew, Saesi Tsaedaemba, Hintalo Wajirat and Alamata), which were randomly selected as a stratified sample of one District per Zone, as shown in Fig 1. The sampled districts included a total of 183,286 households, with a total population of 843,115, covering around 20% of the total population of rural Tigray. Of these, 166,515 were women of reproductive age (WRA), defined as 15–49 years, representing 19% of all women of reproductive age in rural Tigray.

### Study design, sample size and sampling procedure

A community-based case-control study was designed, with geographical matching at the lowest administrative level (*tabia*), to assess the individual-level risks for maternal death while controlling for wider geographic variations. A case of maternal death was defined as a woman of reproductive age (15–49 years old) who died during pregnancy, childbirth or puerperium due to maternity-related causes. Controls were defined as women of reproductive age group (15–49 years old) who delivered a live child during the reference period and who were alive six weeks postpartum.

Sample size was calculated using Epi Info Version 3.5.1 with the following assumptions: 95% confidence, 80% power, proportion of exposure among controls to key parameters as 20%, odds ratio of 2.5, a case to control ratio of 4:1 and a 10% contingency for non-responses. This gave a total sample size of 62 cases and 248 controls. Since the one-year survey period from May 2012 to May 2013 previously described [5] included 51 maternal deaths, this study included those 51 as cases, and recruited a further 11 cases in the three months following the one-year survey period.

A census of all households in the six selected Districts was conducted in mid–2013 to identify all births and deaths among women aged 15 to 49 years. The causes of death were surveyed using the 2012 WHO VA instrument [14], processed using the InterVA-4 model [15], and all maternal deaths were included in the study as cases. A list was then drawn up of all the living women in the same reproductive age group who gave birth during the study period and lived in the same *tabia* as each case. This was used as a sampling frame for the selection of controls. Four controls were selected for each case, using simple random sampling, from these lists.

### Data Collection procedure

Data were collected by trained Maternal and Child Health experts, responsible for the maternal and child health programmes at district level, with the help of health extension workers as community guides, using a structured questionnaire developed by reviewing similar studies and different relevant guidelines and protocols. The questionnaire was developed in English and then translated into the local language (Tigrigna). Adult respondents who were caregivers at the time of death of the mother were used to collect information about all cases. The information about controls was collected from the controls themselves. Supervisors were trained by the principal investigator for one day and the data collectors were trained by the supervisors for three days on the details of data collection tool, interviewing techniques, the importance of data quality and research ethics. Written consent was obtained from all the controls and adult respondents who were caregivers at the time of death of the mother. The consent was approved by the Institutional Review Board of Mekelle University

### Data processing and analysis

Data were analysed using Stata 11. Bivariable logistic regression was used to analyse relationships between maternal death and independent variables, with crude odds ratios and 95% confidence intervals estimated for each parameter. A series of nine parameters relating to husbands’ involvement with maternity were appreciably inter-correlated, and consequently a score for husbands’ involvement was developed, based on the number of positive responses out of the nine items. This score was dichotomised as above or below the median number of positive responses and used as a single variable in the further analyses. Multivariable logistic regression was used to build an overall model from the factors that were significantly associated with maternal mortality on a bivariate basis, giving adjusted odds ratios and 95% confidence intervals. The dataset on which these analyses are based is available on request from the Corresponding Author at hgodefay@yahoo.com.

## Ethics

Ethical approval for the study was granted by the Institutional Review Board (IRB) of the College of Health Sciences of Mekelle University, Ethiopia. Written informed consent was sought from all living mothers (controls) and relatives of the deceased mothers after explaining the risks and benefits of the study, and all participants had the opportunity to withdraw at any stage. The consent procedure was approved by the Institutional Review Board of Mekelle University.

## Results

A total of 310 women (62 cases and 248 controls) participated in this study, and all agreed to do so. Half of the women were in the 25–34 year age group. The majority were of Tigrean ethnicity (97.1%), and married or partnered (85.8%). Similarly, the majority (80.0%) were farmers and had no formal education (80.7%). Non-membership of the Women’s Development Army (WDA), which is an important social movement in Ethiopia intended to improve maternal and child health, showed an overall significant association with the risk of maternal death (odds ratio 2.12, 95% CI 1.16–3.89), although women involved as leaders in the WDA also had a lower risk. These and other background characteristics of the women are shown in Table 1. It should be noted that, apart from WDA membership, none of the background characteristics in Table 1 was significantly associated with maternal death.

**Table 1:** Socio-demographic and cultural characteristics of 62 rural Tigrean women who died of maternal causes and 248 controls (who had given birth in the study period May 2012 to August 2013 and lived in the same communities as the maternal deaths).

		Maternal Status		Crude Odds Ratio (95% CI)
Characteristics	Values	Alive N (%)	Died N (%)	
Age group *(matched)*	15–24 years	63 (25.4)	17 (27.4)	-
	25–34 years	126 (50.8)	28 (45.2)	
	35+ years	59 (23.8)	17 (27.4)	
Residence	Semi-urban	64 (25.8)	13 (21.0)	
	Rural	184 (74.2)	49 (79.0)	1.31 (0.67–2.57)
Ethnicity	Tigrean	240 (96.8)	61 (98.4)	
	Amhara	8 (3.2)	1 (1.6)	0.49 (0.06–4.01)
Religion	Orthodox Christian	239 (96.4)	60 (96.8)	
	Other	9 (3.6)	2 (3.2)	0.89 (0.19–4.20)
Marital Status	Married/partnered	214 (86.3)	52 (83.9)	
	Widowed/divorced	28 (11.3)	8 (12.9)	1.18 (0.51–2.73)
	Never married	6 (2.4)	2 (3.2)	1.37 (0.27–7.00)
Occupation	Farmer	202 (81.5)	46 (74.2)	
	Housewife	37 (14.9)	10 (16.1)	1.19 (0.55–2.56)
	Other	9 (3.6)	6 (9.7)	2.93 (0.99–8.63)
Education	No formal education	199 (80.2)	51 (82.3)	
	Some formal education	49 (19.8)	11 (17.7)	0.87 (0.43–1.80)
Husband’s education	No formal education	165 (66.5)	40 (64.5)	
	Some formal education	83 (33.5)	22 (35.5)	1.09 (0.61–1.96)
Husband’s occupation	Farmer	217 (87.5)	55 (88.7)	
	Other	31 (12.5)	7 (11.3)	0.89 (0.37–2.13)
Responsibility in Women’s Development Army	WDA member only	165 (64.5)	38 (66.1)	
	WDA leadership	32 (12.9)	2 (3.2)	0.27 (0.06–1.18)
	Not WDA member	51 (22.6)	22 (30.7)	1.87 (1.02–3.45)
Monthly income	<250 ETB	97 (39.1)	26 (41.9)	
	251–500 ETB	104 (41.9)	21 (33.9)	0.75 (0.40–1.43)
	501–1,000 ETB	36 (14.5)	10 (16.1)	1.04 (0.45–2.36)
	>1,000 ETB	11 (4.4)	5 (8.1)	1.70 (0.54–5.31)

A series of nine questions were included on topics relating to the involvement of women’s husbands or partners in relation to maternity. These items are shown in Table 2, and with one exception were all significantly related to the risk of maternal death. Not surprisingly, there were many inter-correlations in the responses, given the similarities between the issues explored, and so the overall support of the husband was characterised in a score representing the number of positive responses to the nine issues. Since there was no reason to suppose that this score had a linear relationship with the risk of maternal death, it was dichotomised as below and above the median value (0 to 6 and 7 to 9 respectively). This new variable was also significantly associated with maternal death (bivariable odds ratio 2.37, 95% CI 1.32–4.24), and was used to represent husbands’ involvement in further stages of the analyses.

**Table 2:** Husbands’ involvement in maternity issues for 62 rural Tigrean women who died of maternal causes and 248 controls (who had given birth in the study period May 2012 to August 2013 and lived in the same communities as the maternal deaths). Husband’s involvement score is based on the number of positive responses to the nine questions.

Husband’s Involvement Questions		Maternal status		Crude Odds Ratio (95% CI)
		Alive N (%)	Died N (%)	
Did her/your husband prepare transportation to go to a health facility during ANC/delivery services?	Yes	158 (63.7)	27 (43.6)	
	No	90 (36.3)	35 (56.5)	2.27 (1.29–4.00)
Did her/your husband go with you/her to a health facility during ANC/delivery?	Yes	148 (59.7)	24 (38.7)	
	No	100 (40.3)	38 (61.3)	2.34 (1.32–4.15)
Did her/your husband encourage you/her to get ANC/delivery services?	Yes	172 (69.4)	28 (45.2)	
	No	76 (30.7)	34 (54.8)	2.75 (1.56–4.85)
Did her/your husband remind you/her of the time for ANC or of delivery?	Yes	152 (61.3)	23 (37.1)	
	No	96 (38.7)	39 (62.9)	2.68 (1.51–4.77)
In previous pregnancies, did her/your husband go with you/her to a health facility during ANC/delivery?	Yes	137 (55.2)	26 (41.9)	
	No	111 (44.8)	36 (58.1)	1.71 (0.98–3.00)
Is her/your husband the main decision maker for when and where to get ANC/delivery services?	Yes	136 (54.8)	20 (32.3)	
	No	112 (45.2)	42 (67.7)	2.55 (1.42–4.59)
Have you/the mother ever discussed issues regarding family planning with your/her husband?	Yes	154 (62.1)	23 (37.1)	
	No	94 (37.9)	39 (62.9)	2.78 (1.56–4.94)
Have you/the mother ever discussed issues regarding to ANC with your/her husband?	Yes	170 (68.6)	26 (41.9)	
	No	78 (31.5)	36 (58.1)	3.01 (1.70–5.34)
Have you/the mother ever discussed issues regarding delivery, including the place of delivery, with your/her husband?	Yes	165 (66.5)	26 (41.9)	
	No	83 (33.5)	36 (58.1)	2.75 (1.56–4.86)
**Husband’s involvement score**	**Above median (7–9)**	**136 (54.8)**	**21 (33.9)**	
	**Below median (0–6)**	**112 (45.2)**	**41 (66.1)**	**2.37 (1.32–4.24)**

The health characteristics and decision-making power of the women were ascertained in a further series of questions, summarised in Table 3 in relation to the risk of maternal death. A number of these parameters were significantly associated with the risk of maternal death on a bivariable basis (previous pregnancy-related complications, any previous medical illness, the non-use of contraceptives, below-median number of antenatal care visits, non-involvement in health-care decision making).

**Table 3:** Health related characteristics and women’s decision making power for 62 rural Tigrean women who died of maternal causes and 248 controls (who had given birth in the study period May 2012 to August 2013 and lived in the same communities as the maternal deaths).

Characteristics	Values	Maternal Status		Crude Odds Ratio (95% CI)
		Alive N (%)	Died N (%)	
***Medical history*:**				
History of abortion	Yes	26 (10.5)	6 (9.7)	
	No	221 (89.5)	56 (90.3)	1.10 (0.43–2.80)
Previous pregnancy-related complications	No	232 (93.9)	52 (83.9)	
	Yes	15 (6.1)	10 (16.1)	2.97 (1.27–6.99)
Previous Caesarean section	No	245 (98.8)	59 (95.2)	
	Yes	3 (1.2)	3 (4.8)	4.15 (0.82–21.1)
Any previous medical illnesses	No	237 (95.6)	47 (75.8)	
	Yes	11 (4.4)	15 (24.2)	6.88 (2.97–15.9)
Ever used contraceptives before the last pregnancy	Yes	156 (62.9)	21 (33.9)	
	No	92 (37.1)	41 (66.1)	3.31 (1.84–5.95)
***This pregnancy*:**				
Gravidity	First pregnancy	29 (11.9)	11 (17.7)	
	2–4 pregnancies	137 (55.2)	25 (40.3)	0.48 (0.21–1.09)
	5+ pregnancies	82 (33.1)	26 (41.9)	0.84 (0.37–1.90)
Parity	Undelivered or first	48 (19.4)	15 (24.2)	
	2–4 deliveries	130 (52.4)	27 (43.6)	0.66 (0.33–1.36)
	5+ deliveries	70 (28.2)	20 (32.3)	0.91 (0.43–1.96)
Number of antenatal care visits	Above median (3+)	136 (54.8)	24 (38.7)	
	Below median (0–2)	112 (45.2)	38 (61.9)	1.92 (1.09–3.40)
Involved in health care decision making	Yes	220 (88.7)	48 (77.4)	
	No	28 (11.3)	14 (22.6)	2.29 (1.12–4.68)
Involved in major expense decision making	Yes	210 (84.7)	46 (74.2)	
	No	38 (15.3)	16 (25.8)	1.92 (0.99–3.74)

In Table 4 we show the results from a multivariable logistic regression model which included all the factors associated with maternal death on a bivariable basis (WDA membership, husbands’ involvement score, and the five significant factors identified in Table 3). In the multivariable analysis, significant associations with the risk of maternal death were maintained by non-membership of the WDA, below-median score for husbands’ involvement, previous medical illness, and not having used contraception before the pregnancy.

**Table 4:** Bivariable and multivariable analysis of factors significantly associated with maternal mortality for 62 rural Tigrean women who died of maternal causes and 248 controls (who had given birth in the study period May 2012 to August 2013 and lived in the same communities as the maternal deaths).

Factor	Value	Crude Odds Ratio (95% CI)	Adjusted Odds Ratio (95% CI)
***Medical history*:**			
Previous pregnancy-related complications	No	ref	ref
	Yes	2.97 (1.27–6.99)	1.63 (0.57–4.61)
Any previous medical illnesses*	No	ref	ref
	Yes	6.88 (2.97–15.9)	5.58 (2.17–14.30)
Ever used contraceptives before the last pregnancy*	Yes	ref	ref
	No	3.31 (1.84–5.95)	2.58 (1.37–4.85)
***This pregnancy*:**			
Number of antenatal care visits	Above median (3+)	ref	ref
	Below median (0–2)	1.92 (1.09–3.40)	1.67 (0.88–3.15)
Involved in health care decision making	Yes	ref	ref
	No	2.29 (1.12–4.68)	2.12 (0.94–4.78)
***Personal factors*:**			
Member of Women’s Development Army*	Yes	ref	ref
	No	2.12 (1.16–3.89)	2.07 (1.04–4.11)
Husband’s involvement score*	Above median (7–9)	ref	ref
	Below median (0–6)	2.37 (1.32–4.24)	2.19 (1.14–4.18)

*Variables significantly associated with maternal death (p < 0.05) in bivariable and multivariable analyses

## Discussion

Although maternal mortality is clearly a complex and multifactorial problem, in this study we were able to identify some important individual determinants of the risks for maternal death, while controlling for extrinsic factors such as geography and access to services (by matching cases and controls within local areas). Of the four factors that emerged with statistical significance, two related to individual social factors (membership of the WDA and husbands’ involvement) and two to personal medical history (previous illnesses and non-use of contraceptives). All four factors can therefore potentially lead to public health actions—the social ones through health education messaging, and the medical history ones as key risk flags to pick up through antenatal care.

One of the most important findings of this study was the effect of husbands’ involvement in maternity on reducing the risk of maternal death. This indicates the importance of husbands’ involvement in discussing maternity services with their wives, providing material and moral support in seeking maternity services, and accompanying mothers to maternal health care services. A study conducted in Ethiopia pointed out that women’s access to health services is frequently determined by their husbands, which can serve to inhibit women’s utilisation of formal health resources, in addition to the expectation that the husband is widely expected to be available for decisions and financial support if complications arise. [11]

Discussion among couples regarding maternal health services may indicate husbands’ concern for women’s health, enhancing women’s participation (empowerment) in the decision-making process, which might in return result in better health outcomes. Studies in Nepal found that women whose husbands show concern in pregnancy were more likely to utilize reproductive health services. [16] In Bangladesh, interventions targeted at men were found to improve their awareness of reproductive health issues. [17] In addition, the 2011 Ethiopian Demographic and Health Survey (EDHS) showed that contraceptive use, access to antenatal care and delivery assistance from a skilled provider increases with women’s empowerment. [6]

Though the Ethiopian male role during childbirth may be primarily as a decision-maker, certain communities value the emotional and practical support offered by husbands. A qualitative study conducted in Ethiopia pointed out that husbands often take a supportive role during pregnancy, childbirth and the postpartum period, and the degree of their material and moral support can be a significant determinant of maternal and neonatal health outcomes. [18] Studies in Uganda and Bangladesh revealed that women were more likely to have better outcomes when their husbands got directly involved in maternal health care by attending ANC visits and supported their wives during pregnancy. [19,20] A detailed analysis of the Kenya Demographic and Health Survey (KDHS) showed that women whose husbands attended at least one ANC visit were more likely to have skilled birth attendance than those whose husbands did not attend any ANC visits. [21]

To date, most of the efforts to address maternal health outcomes and their determinants have focused primarily on the women themselves, so this finding on the importance of husbands’ involvement necessitates new approaches to maternal health interventions. Cognisant of the importance and the lack of existing effort to involve male partners, planners of maternal health programmes at various levels need to develop innovative approaches that promote male involvement in reproductive health services.

Another important finding, which has been quantified here for the first time, is the reduced risk of maternal death associated with being a member of the WDA, a women-centred voluntarily organised group in Ethiopia, which comprises groups of up to 25–30 women residing in the same neighbourhood. These groups are further divided into smaller groups of six members, commonly referred as one-to-five networks. Mothers who were members of the WDA were found to be less likely to experience maternal death as compared to non-members. Through its organised voluntary groups of women, the WDA is conceptualised as a way to create a demand for health, wellness and improved access to health care services, and it enables efficient communication and mobilisation. The network meets daily and the subgroup leaders have an additional meeting every three days. The WDA is considered critical to the successful implementation of the Health Extension Programme (HEP). This is an innovative community-based strategy to deliver preventive and health promoting services and selected high impact curative interventions at community level, with particular emphasis on improving uptake of critical maternal and new-born health services. In each locality it is run by two female Health Extension Workers (HEW), who are graduates of a one-year certificate programme, after completing 10^th^ grade in school. Some of their contributions to maternal health services include encouraging mothers to use family planning services, mobilizing pregnant mothers to attend antenatal care, following up pregnant mothers to promote skilled delivery, supporting the community, and providing needs-based psychological and moral support to mothers. This is consistent with a systematic review of groups similar to the Ethiopian WDA in Bangladesh, India, Malawi and Nepal which showed significant reductions in maternal mortality. [22] Since the concept of WDA is new, and particular to Ethiopia, its contribution to reducing maternal mortality has not yet been systematically assessed and documented. Nevertheless, these initial findings are encouraging.

Another important finding is that the presence of pre-existing medical illness was significantly associated with increased maternal mortality. This has also been reported in other studies in developing countries. [8,23–25] This is related to the effect of indirect obstetric causes on maternal mortality. A community-based study of the magnitude and causes of maternal mortality in the same study areas showed that indirect causes accounted for 27% of all maternal deaths. The most common indirect obstetric causes were anaemia (12%), tuberculosis (10%), HIV/AIDS (2%) and malaria (2%) [5]. It has been suggested that integrating the care of women and children with other services is an efficient and cost-effective route to success. Thus, stronger links must be built between disease-specific prevention and control programmes (such as for HIV/AIDS, malaria, tuberculosis and non-communicable diseases) and maternal health services (antenatal care, delivery and postnatal care) to prevent as well as to treat these diseases. Particularly, screening and referral of pregnant women who may have anaemia, tuberculosis or HIV/AIDS should be strengthened at community level through Health Extension Workers. In addition, early detection and prompt management of these conditions must be considered for all pregnant women attending maternal health clinics at higher level health facilities. At an individual level, documenting pre-existing disease may need to be given more importance within antenatal care programmes, triggering special follow-up of high-risk women during pregnancy and delivery.

The number of ANC visits was another factor associated with maternal mortality in this study. Women who exceeded the median number of visits, amounting to 3 or more in this dataset, experienced lower maternal mortality. This result is in line with other similar studies elsewhere [12,23,24]. While antenatal care programmes may not be the entire solution to reducing maternal mortality, our findings showed that making more antenatal care visits was beneficial, and may also lead towards a greater uptake of skilled delivery attendance. A nationally representative survey in Kenya showed that the number of ANC visits was significantly associated with skilled delivery. [21] Nevertheless, only 19.1% of pregnant women from rural areas in Ethiopia nationally reported making four or more ANC visits [6]. It was not, however, possible to determine the exact relationship between the number of antenatal care visits and the risk of maternal death from this relatively small study, and we have no evidence that there was a linear relationship; simply that more visits were advantageous.

Women who had ever used contraceptives were also at considerably reduced risk of maternal mortality in this study, though the mechanism behind this is not clear. It could simply be that those making use of contraceptive services were more aware of reproductive health issues and more keen to assert control over their reproductive pattern. This is consistent with a facility-based case control study conducted in Kenya. [25] The use of family planning might contribute to maternal survival as a result of increased birth spacing. [10,21] A study in India revealed that maternal mortality nearly doubled with birth intervals under two years. [26] In any case, efforts to increase family planning utilisation should be strengthened in the interests of increasing maternal survival, and women who have never used contraceptives might be identified in antenatal care as being at particular risk, even if the risk mechanism is not clear.

The study was limited to some extent by the relatively small number of maternal deaths that occurred in the six randomly selected Districts, with the result that only relatively large effects could be identified. A number of important factors nevertheless emerged. Since no sampling other than the random selection of the Districts was involved, it is reasonable to suppose that the cases and controls were representative of the Region. Controls were selected from the same local areas as cases to ensure that observed differences did not relate to variations in local geographic factors; similarly age group was matched to ensure that findings were controlled for age. Consequently no conclusions about the effects of geography or age group could be drawn here, though these factors have been analysed previously. [5]

## Conclusions

Husbands’ involvement in maternal health care, women’s participation in the WDA, a lack of pre-existing medical illness, and ever using contraceptives were all independently and significantly associated with lower risks of maternal mortality.

Mechanisms to promote and ensure male involvement in all maternal health care services therefore need to be promoted, as well strengthening activities of the WDA. Stronger links must be built between disease-specific prevention and control programmes and maternal health services to avert indirect causes of maternal mortality. A streamlined process of identifying individual risks relating to other diseases at early antenatal care visits, and flagging those risks throughout pregnancy and delivery, needs to be implemented. Similarly women who have never used contraceptives could be identified in early antenatal care and specifically followed up, even if the precise mechanism of the risks pertaining to this group may not be clear.

These findings are in many ways consistent with what might be expected, and they clearly support a recognition of the basic need to provide the best possible standards of maternal care for every woman throughout pregnancy and delivery. However, we have shown that there are tractable issues associated with maternal deaths in Tigray which need to be followed up in order to avoid future deaths.
